# Managing Abnormal Uterine Bleeding With Norethisterone Acetate: An Evidence-Based Review and Expert Insights

**DOI:** 10.7759/cureus.106257

**Published:** 2026-04-01

**Authors:** Hema Divakar, Manjula Anagani, Parikshit Tank, Sukanta Misra, Jayam Kannan, Girija Wagh, Mitra Saxena, Farendra Bhardwaj, Jayita Chakrabarti, Onkar C Swami, Kamal Kumar, Neeraj Kumar

**Affiliations:** 1 Obstetrics and Gynaecology, Divakars Specialty Hospital, Bengaluru, IND; 2 Woman and Child Institute, CARE Hospitals, Hyderabad, IND; 3 Obstetrics and Gynaecology, Ashwini Maternity and Surgical Hospital, Mumbai, IND; 4 Gynaecology, Vivekananda Institute of Medical Sciences, Ramakrishna Mission Seva Pratishthan, Kolkata, IND; 5 Obstetrics and Gynaecology, Garbba Rakshambigai Fertility Centre (GFC) India, Chennai, IND; 6 Obstetrics and Gynaecology, Bharati Vidyapeeth Medical College, Pune, IND; 7 Obstetrics and Gynaecology, Shri Ashwini Saxena Hospital, Rewari, IND; 8 Obstetrics and Gynaecology, Mahatma Gandhi Medical College and Hospital, Jaipur, IND; 9 Obstetrics and Gynaecology, Manipal Hospital, Salt Lake, Kolkata, IND; 10 Medical Services, Alembic Pharmaceuticals Ltd, Mumbai, IND

**Keywords:** abnormal uterine bleeding, controlled-release formulations, expert consensus, expert opinion, norethisterone acetate

## Abstract

Abnormal uterine bleeding (AUB) is a commonly presented gynaecological disorder that significantly affects the physical, emotional, and social well-being of a woman. Norethisterone acetate (NETA), a synthetic progestin, is widely utilized for managing AUB in view of its proven efficacy and favourable safety profile. Despite its extensive use, optimal dosing, duration, and patient-specific considerations in Indian women remain undefined. This expert opinion employed a two-pronged approach to assess the role of NETA in managing AUB in India. A comprehensive literature review was conducted to assess the evidence on efficacy, safety, pharmacokinetics, and formulation benefits, followed by four expert advisory board meetings held across India involving nearly 50 practicing experts. During expert advisory meetings, the current literature was discussed, and expert opinions were developed. The findings suggest that NETA is an effective option for stabilizing abnormal uterine bleeding, particularly during perimenopause, offering rapid symptom control, and well-tolerated and high patient satisfaction across multiple indications, including heavy menstrual bleeding, endometrial hyperplasia, and adenomyosis. Controlled-release formulations of NETA demonstrated pharmacokinetic equivalence to immediate-release regimens and comparable efficacy with the added advantage of improved adherence. Experts highlighted the importance of individualized dosing based on patient characteristics, such as body mass index, bleeding severity, and comorbidities.

## Introduction and background

Abnormal uterine bleeding (AUB) significantly affects multiple aspects of a woman’s life, leading to physical discomfort, emotional distress, and disruption of daily activities. It can also impair daily functioning, social engagement, and the overall quality of life, underscoring the importance of effective management strategies. Globally, approximately one in three women experiences AUB. In India, the prevalence ranges from 3% to 30%, with notable regional variations [1].

A nationwide survey of 141 gynaecologists reported that 32.72% of women seek medical support due to AUB-related symptoms. The survey also highlighted that women of reproductive age were the most affected, with heavy menstrual bleeding (HMB) being the predominant concern [2]. An observational study from northeastern India reported an AUB prevalence of 20.48%, with the highest incidence among women aged 41-45 years, and leiomyoma identified as the leading cause [3]. Similarly, studies from other regions of the country recorded a prevalence of 18.30%, while in Shimla, Himachal Pradesh, it was documented to be 17.09%, with HMB consistently reported as the most common presentation [4,5].

Multiple medical and surgical treatment options are available for AUB and HMB, each of which has specific benefits and limitations. Medical management is the first-line approach for most patients to avoid risks associated with surgery and preserve fertility. Combined oral contraceptives effectively regulate menstrual cycles but may increase the risk of thromboembolism and other complications, and are relatively or absolutely contraindicated in existing vascular disease and in smokers over 35 years of age. The levonorgestrel intrauterine system (LNG-IUS) is reliable in managing HMB and providing contraception, although it may cause intermenstrual bleeding and mood changes, and in the presence of fibroids, is of limited usefulness. Progesterone-only pills are suitable for women who cannot use estrogen but are less effective for HMB. Gonadotropin-releasing hormone (GnRH) agonists are useful for fibroid-related HMB but are recommended only for short-term use due to adverse effects such as hot flashes and bone density loss. Injectable progestogens are effective contraceptives and can induce amenorrhea in many women with HMB; however, they may lead to weight gain and bone loss. Oral progestogens are commonly used to induce withdrawal bleeding in acute AUB. While long-term use may cause endometrial atrophy, these agents remain valuable for short-term symptom control [1,6,7].

Norethisterone (NET), also known as norethindrone, a synthetic analog of natural progestogens, is widely used to manage AUB. Norethisterone acetate (NETA), the acetic acid ester of NET, also offers similar benefits. It suppresses endometrial proliferation, thereby regulating unpredictable bleeding and reducing excessive menstrual flow [1]. NET is indicated for the management of abnormal uterine bleeding, endometriosis, and postponement of menstruation. It is also widely used for contraception either in monotherapy or in combination with ethinyl estradiol (EE). NET is considered cost-effective and generally well tolerated, with minimal adverse effects [1].

Despite the widespread clinical use of NETA/NET for AUB, there remains a lack of consensus on the optimal dosing strategies, treatment duration, and practical considerations across diverse Indian patient profiles, such as those with obesity, endometrial hyperplasia, or perimenopausal symptoms. This manuscript addresses current gaps by synthesizing published evidence with real-world clinical perspectives on NETA/NET. It presents evidence-based recommendations for use, underscores practical considerations and unmet clinical needs, and delineates research priorities aimed at optimizing abnormal uterine bleeding management and patient outcomes.

## Review

Methodology

**Figure 1:**
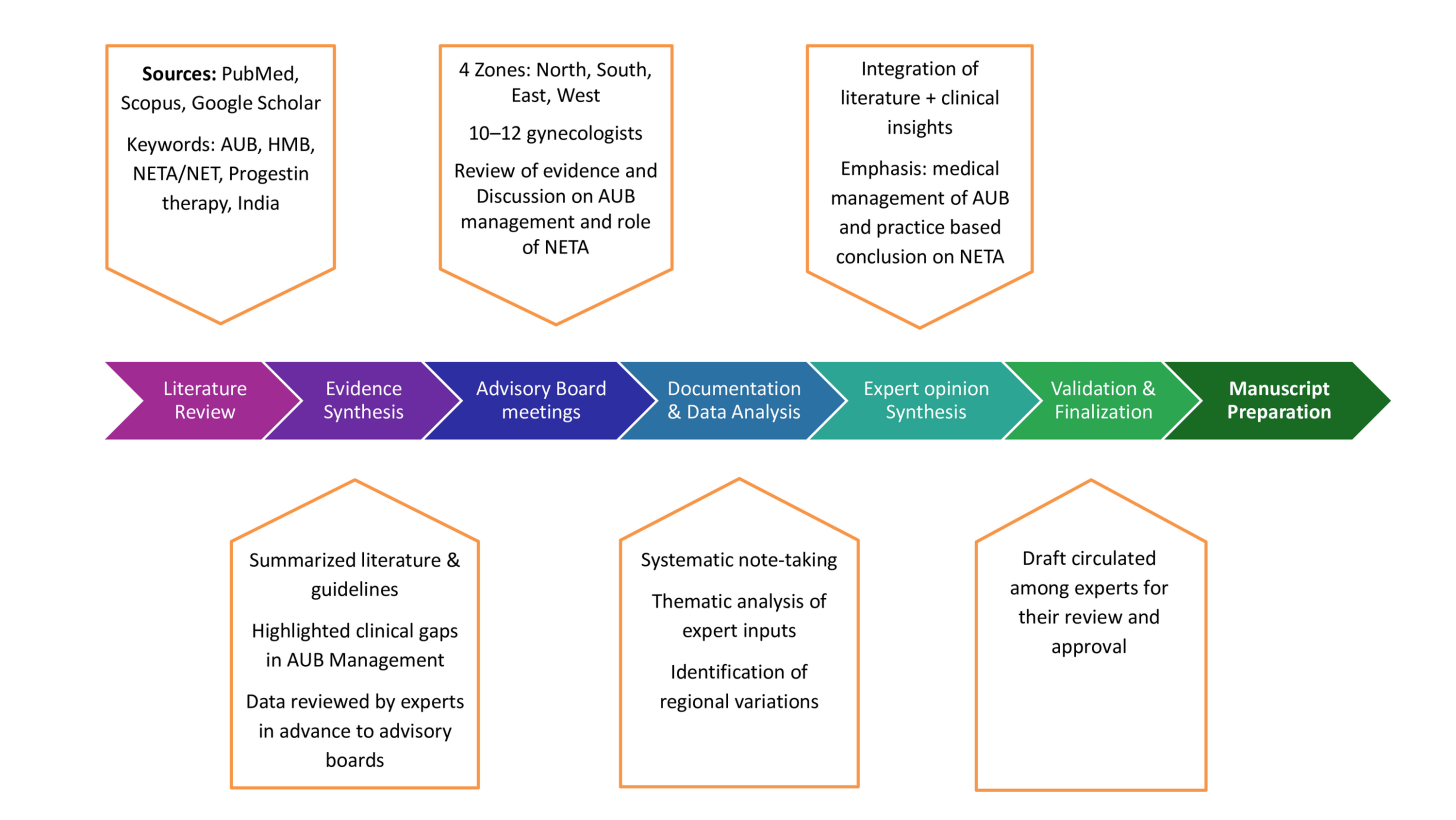
Methodology AUB: abnormal uterine bleeding; NET: norethisterone; NETA: norethisterone acetate; HMB: heavy menstrual bleeding Image created by authors using Microsoft PowerPoint (Microsoft Corporation, Redmond, USA)

This expert opinion-based review article employed a two-pronged approach to assess the role of NETA in managing AUB in India. First, a comprehensive literature review was conducted to collate and analyse existing evidence on NETA's efficacy, safety, and clinical application of NETA in AUB management. Databases such as PubMed, Scopus, and Google Scholar were searched using specific keywords, including "norethisterone acetate", "norethisterone", "abnormal uterine bleeding", "NETA", "NET", "AUB management", "India", "progestin therapy", and "clinical outcomes". Second, the relevant literature was distributed among the expert panel in advance and thoroughly reviewed before the meetings. To gather real-world clinical insights, four regional advisory board meetings were conducted across India in the North, South, East, and West regions. Each meeting included 10-12 leading experts with experience in managing AUB and using NETA. Discussions during the meetings were meticulously documented, and the qualitative data were analysed to extract viewpoints, regional practice patterns, and emerging trends in AUB management. The expert opinions derived from these comprehensive discussions are presented in the following sections, emphasizing the practical implications and clinical relevance to current practice (Figure 1).

Expert discussion

Risk Factors, Screening, and Classification of AUB

Newly identified risk factors and shifting patterns are transforming the clinical landscape of AUB. These include early onset of menarche, metabolic disorders, hormonal usage including emergency contraception, endocrine and thyroid disorders, etc. [8,9]. Notably, women with metabolic syndrome have been shown to be 15 times more likely to develop AUB compared to those without the condition [10]. Premenopausal women >40 years of age with endometrial thickness >13 mm, body mass index (BMI) >25 kg/m^2^, and hypothyroidism are at a higher risk of developing endometrial hyperplasia and endometrial cancer [11]. During the menopausal transition, AUB also impacts women’s health, with factors like age, BMI, and intrauterine device (IUD) placement affecting the severity of AUB [12]. Therefore, a thorough evaluation is essential, which should include a detailed menstrual history covering age at menarche, date of the last menstrual period, cycle frequency, duration, regularity, flow volume, clot size, and the frequency of sanitary product use [13].

For standardized evaluation and classification, the International Federation of Gynecology and Obstetrics (FIGO) developed the PALM-COEIN system. This framework categorizes AUB etiologies into structural causes, including Polyp, Adenomyosis, Leiomyoma, and Malignancy/hyperplasia (PALM), and non-structural causes, including Coagulopathy, Ovulatory dysfunction, Endometrial disorders, Iatrogenic factors, and Not otherwise classified conditions (COEIN). The adoption of this system enhances diagnostic accuracy and informs evidence-based management strategies for women of reproductive age [14].

Expert Opinion

Lifestyle and metabolic factors, including early menarche, obesity, sedentary behavior, use of emergency contraception, and endocrine disorders, are increasingly impacting the epidemiology of abnormal uterine bleeding. Proactive screening and preventive strategies in younger women are essential to facilitate early detection and management of AUB. Particular emphasis should be placed on metabolic assessment, as women with metabolic syndrome demonstrate a significantly elevated risk of developing AUB. The FIGO PALM-COEIN classification system should be adopted universally as the standardized framework for evaluating AUB cases.

Overview of NETA

**Figure 2:**
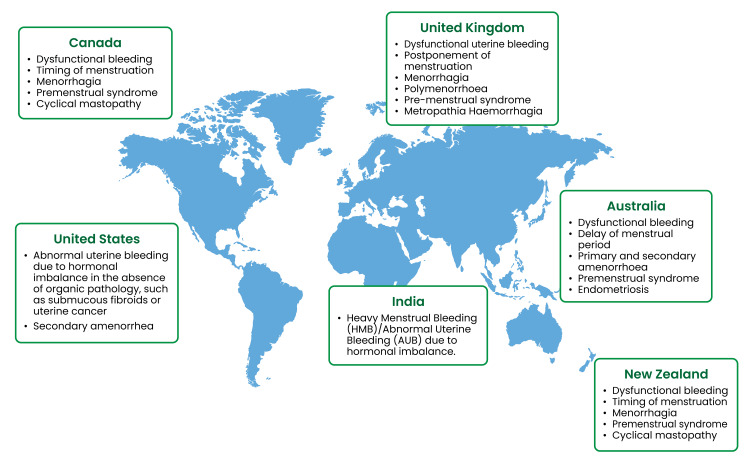
Current status of NET/NETA worldwide NET/NETA: norethisterone/norethisterone acetate The world map was downloaded from Freepik (Málaga, Spain) and edited using Adobe Illustrator (Adobe Inc., San Jose, USA) Source: References [16-20]

NET was the first-generation progestogen developed in 1950, and it is the first orally active progesterone used in oral contraceptives. It exhibits strong progestogenic activity along with tissue-specific androgenic, antigonadotropic, anti-estrogenic, and estrogenic properties owing to its high receptor-binding affinity (Table 1; Figure 2) [1,15].

**Table 1:** Current approved indications of NET/NETA worldwide NET/NETA: norethisterone/norethisterone acetate

UK [16]	US [17]	Australia [18]	New Zealand [18]	Canada [19]	India [20]
Dysfunctional uterine bleeding, postponement of menstruation, menorrhagia, polymenorrhoea, pre-menstrual syndrome, metropathia, Haemorrhagia	Abnormal uterine bleeding due to hormonal imbalance in the absence of organic pathology, such as sub-mucous fibroids or uterine cancer, secondary amenorrhea	Dysfunctional bleeding, delay of menstrual period, primary and secondary amenorrhoea, premenstrual syndrome, endometriosis	Dysfunctional bleeding, timing of menstruation, menorrhagia, premenstrual syndrome, cyclical mastopathy	Dysfunctional bleeding, timing of menstruation, menorrhagia, premenstrual syndrome, cyclical mastopathy	To arrest bleeding in women with heavy menstrual bleeding/abnormal uterine bleeding due to hormonal imbalance, to arrest bleeding in women with dysfunctional uterine bleeding due to hormonal imbalance

Clinical Application and Dosage

Clinically, NET is widely used for managing AUB, endometriosis, and postponing menstruation. Additionally, it is used in contraception, alone or with ethinylestradiol (EE), and as part of hormone therapy (HT) for postmenopausal symptom relief in combination with estrogen. Typical dosages include 10-15 mg/day for therapeutic use, 0.5-1 mg/day for HT, and 0.35 mg/day for contraception (Table 2) [21,22].

**Table 2:** Comparison of effects of NET and other progestins ✓=effective; X=not effective; O=weakly effective Adapted from [23].

Compound	Progesterone	Retroprogesterone	Pregnanes		Norpregnanes	Estranes		Gonanes				Spironolactone derivative
	Progestogen	Dydrogesterone	Medroxyprogesterone (MPA)	Cyproterone acetate	Nomegestrol acetate	Norethisterone (NET)	Lynestrenol	Levonorgestrel	Desogestrel	Gestodene	Dienogest	Drospirenone
Progestogenic	✓	✓	✓	✓	✓	✓	✓	✓	✓	✓	✓	✓
Antigonadotropic	✓	X	✓	✓	✓	✓	✓	✓	✓	✓	✓	✓
Anti-estrogenic	✓	✓	✓	✓	✓	✓	✓	✓	✓	✓	O	✓
Estrogenic	X	X	X	X	X	✓	✓	X	X	X	O	X
Androgenic	X	X	O	X	X	✓	✓	✓	✓	✓	X	X
Anti-androgenic	O	O	X	✓✓	O	X	X	X	X	X	✓	✓
Glucocorticoid	✓	X	✓	✓	X	X	X	X	X	✓	X	X

Pharmacokinetics and Metabolic Conversions

NETA is rapidly absorbed from the gastrointestinal tract and converted to the active form NET through the removal of the acetate group. Post-absorption, NET exhibits partial binding to sex hormone-binding globulin (SHBG; 36%) and predominant binding to albumin (about 61%). The non-protein-bound fraction of NET in circulation is 3%-4% [23]. A well-documented pharmacokinetic property of NETA is its partial hepatic conversion to EE, which is supported by parallel increases in NET/NETA and EE levels. This conversion contributes to NETA’s overall estrogenic activity, with NET’s androgenic effect hypothesized to partially counterbalance EE’s hepatic impact. It is estimated that 1 mg of NETA can yield approximately 6 µg of EE, whereas NET produces lower EE levels owing to reduced conversion efficiency. At therapeutic doses (10-20 mg/day), NETA can produce plasma EE concentrations (58-178 pg/mL) that are comparable to those observed with combined oral contraceptives containing 20-40 µg EE (100-135 pg/mL). The metabolic conversion of NET/NETA to EE may contribute to estrogen-related adverse effects. This conversion is associated with a moderately increased risk of venous thromboembolism (VTE), particularly at higher therapeutic doses, although contraceptive doses generally do not confer this risk; caution is warranted in women with a history of thrombosis. Long-term use has been linked to a slightly elevated risk of breast cancer. Additionally, women with migraine with aura may have an increased risk of ischemic stroke when exposed to EE, although specific evidence for NET/NETA at therapeutic doses remains limited [23].

Effect on Bone Metabolism and Cardiovascular Risks

Bone density is strongly associated with female hormones as estrogen reduces bone remodelling and resorption and maintains bone formation. NET/NETA has the ability to reduce bone resorption markers while maintaining bone formation indices, suggesting a stimulatory effect on bone metabolism. Notably, NETA has a more pronounced impact on bone mineral density (BMD) than medroxyprogesterone acetate (MPA) [15]. Premenopausal women have a lower incidence of cardiovascular disease than men, likely due to estrogen’s favourable effects on lipids, body composition, insulin sensitivity, endothelial function, and coagulation. Postmenopausal HT with progestogens may counteract these benefits. NET’s mixed androgenic-estrogenic activity lowers both high-density lipoprotein (HDL) and low-density lipoprotein (LDL), as well as triglycerides, while preserving the HDL/LDL ratio [24,25]. Overall, this indicates a neutral or potentially favourable effect on cardiovascular risk [21]. NET has a pronounced effect on the endometrium, achieving transformation at 30-60 mg/cycle. In postmenopausal HT, transdermal NET with estradiol prevents endometrial hyperplasia at 140-400 µg/day (sequential) or 170-350 µg/day (continuous) [26].

Role in AUB Management

Among oral progestogens, NETA remains the most clinically used option for AUB because of its proven efficacy, favorable safety profile, and cost-effectiveness [26]. A real-time Indian study reported that a controlled-release (CR) formulation of NETA, administered once daily at a dose of 10 mg, effectively managed AUB symptoms [21]. Furthermore, the guidelines developed by the Federation of Obstetric and Gynaecological Societies of India (FOGSI) include NETA as a recommended option for AUB management [27].

Singh et al. evaluated the pharmacokinetics of norethisterone acetate in six healthy women (25-35 years) with no contraceptive use in the prior year. The plasma half-life of norethindrone acetate was found to be 51.5 hours, compared with 34.8 hours for its main metabolite, norethindrone. The study also reported a low metabolic clearance rate for norethisterone acetate, indicating a prolonged contraceptive effect [28].

Clinical Evidence of NET-CR in the Indian Population

**Figure 3:**
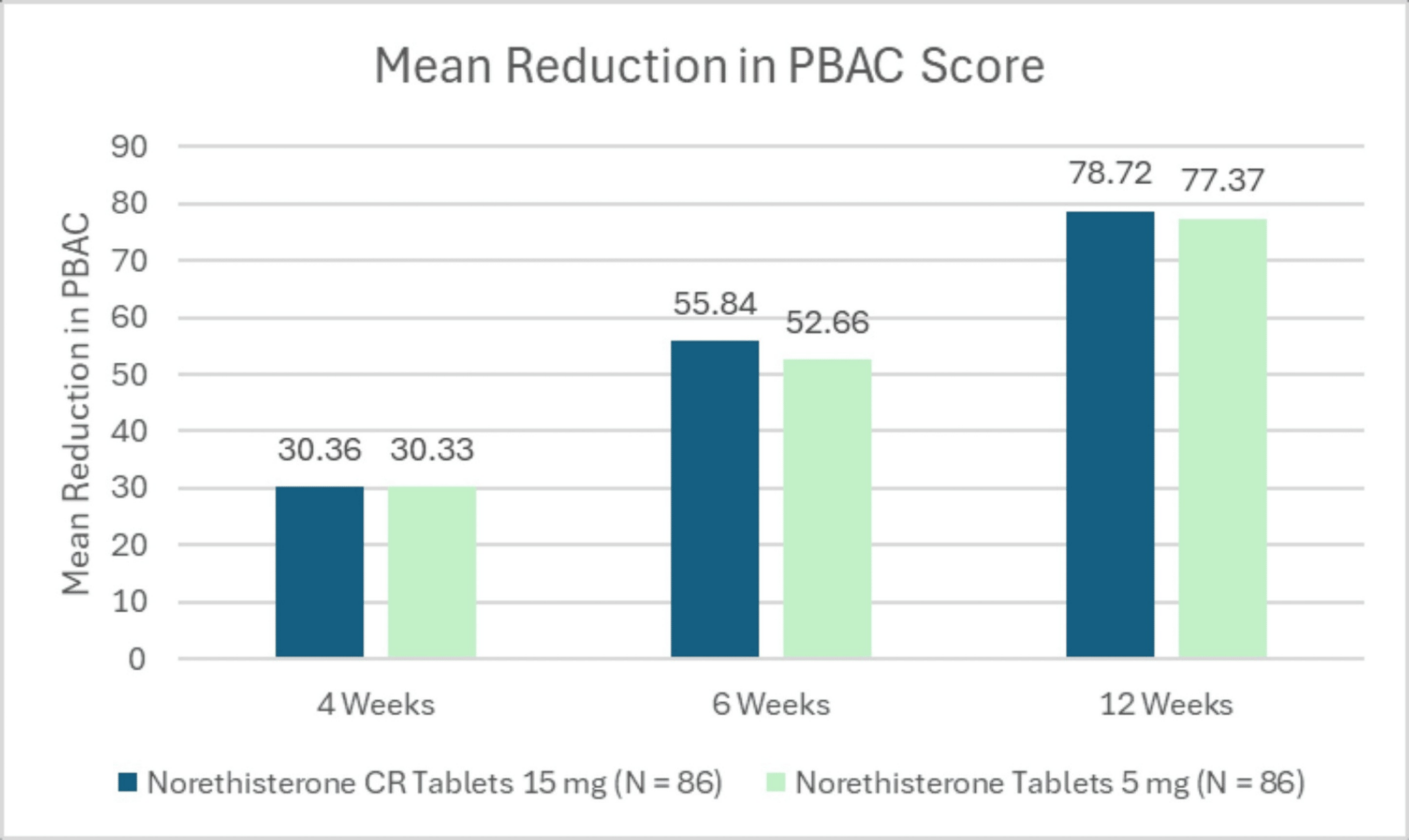
Mean reduction in pictorial blood loss assessment scores PBAC: Pictorial Blood Loss Assessment Chart Source: Original research: Kaushik A, Dwivedi B: A Phase III, Multi-center, Prospective, Randomized, Double Blind, Single Dummy, Active Controlled, Comparative Clinical Study to Evaluate the Efficacy, Safety and Tolerability of Norethisterone CR Tablets 15 mg Versus Norethisterone Tablets 5 mg to Arrest Bleeding in Women With Heavy Menstrual Bleeding (HMB)/Abnormal Uterine bleeding (AUB) due to Hormonal Imbalance; 2019. Image created by authors using Microsoft Excel and Microsoft PowerPoint (Microsoft Corporation, Redmond, USA)

A phase III, multi-center, prospective, randomized, double-blind study was conducted to evaluate the efficacy, safety, and tolerability of NET-CR tablet 15 mg versus norethisterone tablet 5 mg to arrest bleeding in Indian women with HMB/AUB due to hormonal imbalance (Original research: Kaushik A, Dwivedi B: A Phase III, Multi-center, Prospective, Randomized, Double Blind, Single Dummy, Active Controlled, Comparative Clinical Study to Evaluate the Efficacy, Safety and Tolerability of Norethisterone CR Tablets 15 mg Versus Norethisterone Tablets 5 mg to Arrest Bleeding in Women With Heavy Menstrual Bleeding (HMB)/Abnormal Uterine bleeding (AUB) due to Hormonal Imbalance; 2019; CTRI/2019/06/019490). A total of 172 patients were randomized in two arms: 86 patients were enrolled in the norethisterone CR tablet 15 mg arm, and 86 in the norethisterone tablet 5 mg arm. The treatment continued for 84 days with interim follow-ups on day 28±2 (4 weeks) and day 56±2 (8 weeks) from the start of treatment. The end-of-study visit was on day 84±2 (12 weeks) (Figure 3).

The primary endpoint was mean reduction in blood loss during menstruation, assessed using the Pictorial Blood Loss Assessment Chart (PBAC) score; secondary endpoints were percentage responder and changes in bleeding pattern post-treatment compared to baseline. Safety parameters included changes in laboratory parameters, vital parameters, and any reported adverse events.

Results demonstrated that the NET-CR formulation (once daily Crina-NCR, manufactured by Synokem Pharmaceuticals Ltd, India) led to a significant reduction in menstrual blood loss (Figure 3) after three months of treatment. Norethisterone CR tablet 15 mg provided statistically equivalent efficacy in mean reduction in blood loss during menstruation when compared to norethisterone tablet 5 mg. Adverse reactions were observed and documented; however, these events were not statistically significant. Norethisterone CR tablet 15 mg validates the therapeutic equivalence and patient-centric advantages over the norethisterone tablet 5 mg.

In another retrospective analysis across 40 centers in India, the effectiveness of NET-CR in 308 women aged 18-45 years with ovulatory AUB was evaluated. Patients were treated with 10 mg/day of NET-CR for 10 days. The majority (63%) experienced a styptic effect within four hours of administration, 61% reported no breakthrough bleeding, and 70% experienced withdrawal bleeding within 24-72 hours of completing the course. Importantly, no adverse events were reported during the study, indicating that NET-CR was both effective and well-tolerated in this population [21].

A prospective audit conducted at a tertiary care center evaluated the effectiveness of NET in 29 adolescent girls (aged 11-17 years) presenting with abnormal uterine bleeding. NET was administered at 10-30 mg/day based on bleeding severity, duration, and patient weight. Bleeding ceased within a mean of 46 hours, and no serious adverse events were reported. The study concluded that NET is an effective and well-tolerated option for managing acute AUB in adolescents [29]. A hospital-based prospective study evaluated the effectiveness of treating dysfunctional uterine bleeding with endometrial thickness. Of the 60 patients with dysfunctional uterine bleeding, 55% (33/60) had an endometrial thickness <6 mm, 25% had 6-11 mm, and 20% had >11 mm thickness. The authors concluded that women with dysfunctional uterine bleeding showed an association between increased endometrial thickness and BMI [30].

Bioequivalence of NET-CR and IR formulation: Pharmacokinetic analysis demonstrated that the CR formulation of 15 mg of norethisterone (Crina-NCR, once daily) achieved comparable systemic exposure to the conventional immediate-release (IR) formulation (5 mg administered three times daily). The C_max_ (maximum concentration) and the AUC_0-∞_ (area under the concentration-time curve from time zero extrapolated to infinity) of the CR formulation indicated a relative bioavailability of approximately 94.88%. The intra-subject variability (ISCV) remained within acceptable limits (<31%). There were no adverse events or serious adverse events reported in the study. These findings suggest that the CR formulation of norethisterone is bioequivalent to the IR formulation and, therefore, clinically considered as an alternative to the IR formulation (Original research: Kaushik et al.; 2019).

Expert Opinion

For the management of AUB, a daily dose of 10-15 mg NET-CR is recommended, with 15 mg preferred in patients with higher BMI or heavy bleeding. In cases of AUB with increased endometrial thickness, a treatment regimen of 10-15 mg NET-CR daily for approximately three months is advised. Short-term AUB therapy with 10-15 mg NET-CR daily for two weeks, adjusted according to the bleeding pattern, is considered to be effective. NETA provides a reliable and convenient option for menstrual delay in appropriately selected candidates.

Efficacy in Adenomyosis

Adenomyosis is a structural pathology identified in the FIGO PALM-COEIN classification system for abnormal uterine bleeding; it is singled out as it represents a distinct anatomical cause of bleeding in reproductive-aged women [31]. In a study, adenomyosis patients were treated with 5 mg NETA daily using a "three weeks on and one week off" regimen. Significant improvements were observed; dysmenorrhea scores before and after treatment were 62.5 and 11.3, respectively (p < 0.001). Bleeding scores before and after treatment were 28.1 and 8.1, respectively (p < 0.001). Breakthrough bleeding was not reported in any of the patients two months post-therapy [32].

Safety

NETA is effective in rapidly stopping acute heavy bleeding and improving menstrual regularity in both adolescents and adults, with high rates of patient satisfaction and minimal short-term side effects [21,29]. Similarly, in a retrospective review of 176 adolescents, 66% of the participants continued NETA with no side effects (p = 0.001), and no serious adverse events were reported. Among adolescents requiring a norethisterone taper for heavy bleeding, 78.9% experienced complete cessation of bleeding within seven days, suggesting effective symptom control [32]. Long-term use of NETA has demonstrated a favorable safety and tolerability profile in the management of rectovaginal endometriosis [33]. In a five-year retrospective cohort study conducted in Italy, 68.8% (42/61) of women who remained on long-term NETA therapy reported being satisfied or very satisfied, accounting for 40.8% (42/103) of the intention-to-treat (ITT) population. Additionally, 59.2% (61/103) of the participants continued NETA at the five-year follow-up, indicating good long-term adherence and acceptability [34].

Expert Opinion

NETA is generally well-tolerated with minimal side effects, and liver function monitoring is recommended during treatment; any transient elevations in liver enzymes typically resolve upon discontinuation. In patients with cholestasis, shorter treatment durations of NETA are advisable to reduce potential hepatic effects. For the management of AUB, a daily dose of 10-15 mg NET-CR is recommended, with 15 mg preferred in patients with higher BMI or heavy bleeding. In cases of AUB with increased endometrial thickness, a treatment regimen of 10-15 mg NET-CR daily for approximately three months is advised. Short-term AUB therapy with 10-15 mg NET-CR daily for two weeks, adjusted according to the bleeding pattern, is considered to be effective. NETA provides a reliable and convenient option for menstrual delay in appropriately selected candidates.

**Figure 4:**
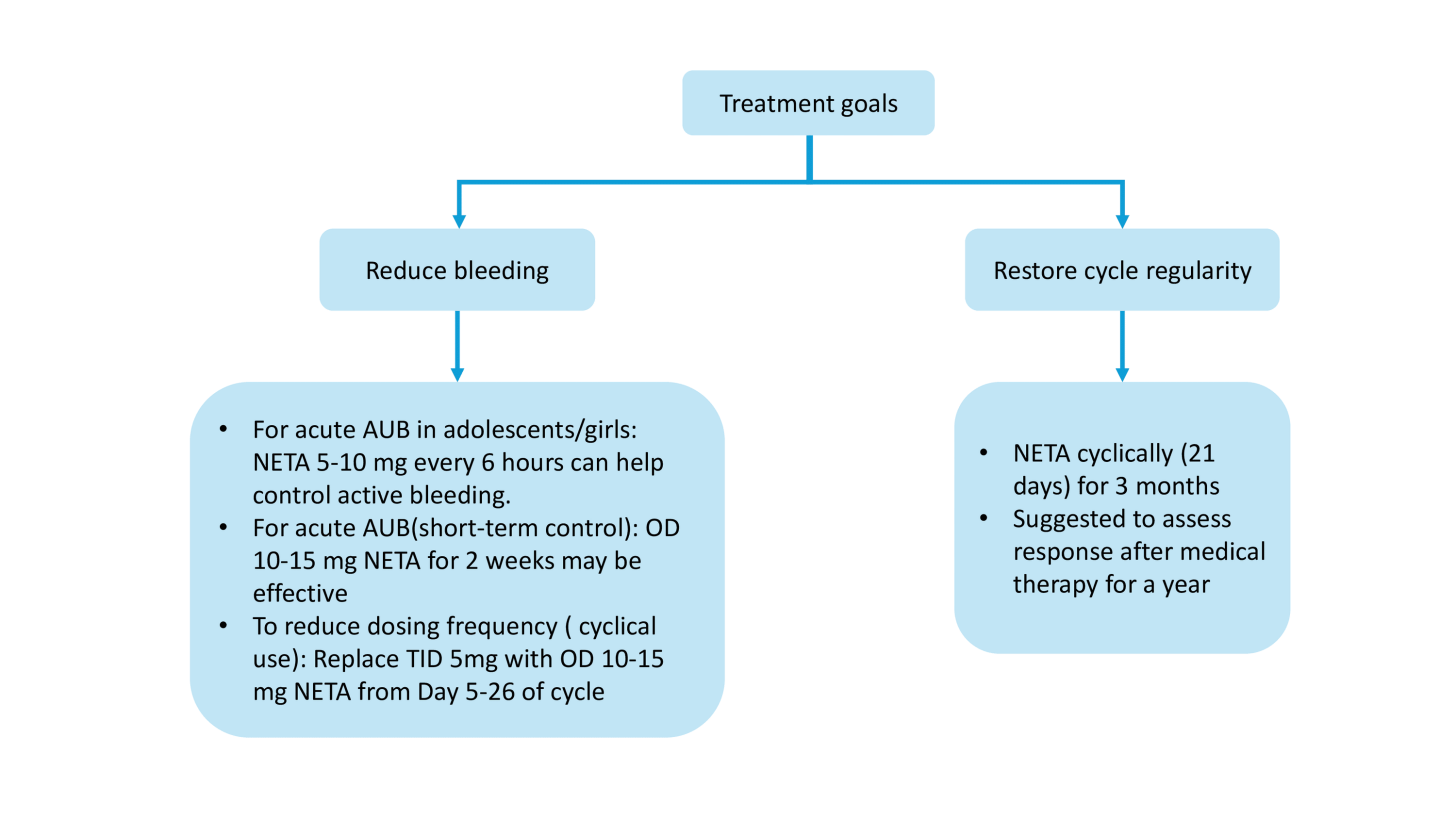
Placement of NETA in the management of AUB AUB: abnormal uterine bleeding; NETA: norethisterone acetate Image created by authors using Microsoft PowerPoint (Microsoft Corporation, Redmond, USA)

To support clinical decision-making, it is important to understand where NETA fits among the various treatment options for abnormal uterine bleeding. Figure 4 illustrates its placement based on clinical evidence, expert consensus, and real-world usage patterns.

Discussion

AUB remains a prevalent condition profoundly affecting women’s physical, emotional, and social well-being. This expert consensus, developed through comprehensive advisory board meetings, leverages real-world clinical insights to complement existing published evidence. Among the strengths of this approach is the integration of diverse expert experiences, which enriches recommendations and ensures their practical applicability across various clinical scenarios.

NETA stands out as a widely used, effective, and well-tolerated treatment option for AUB. It offers rapid bleeding control and high patient satisfaction in multiple contexts, including heavy menstrual bleeding, endometrial hyperplasia, adenomyosis, and adolescent AUB. The consensus underscores the importance of recognizing lifestyle and metabolic factors such as obesity and metabolic syndrome as key contributors and advocates for proactive screening, particularly in younger women, facilitating timely and tailored management.

Another strength of this expert opinion is the endorsement of standardized evaluation tools like the FIGO PALM-COEIN classification system. Adoption of this system supports consistent diagnosis, optimizes treatment decisions, and enhances communication among healthcare providers. Additionally, the use of controlled-release formulations of NETA promotes improved patient adherence and sustained therapeutic effects.

Individualized dosing strategies, generally recommending 10-15 mg daily of NETA with escalation in women with higher BMI or severe bleeding, and dose adjustments based on endometrial thickness or bleeding severity, align with achieving optimal clinical outcomes.

However, as this consensus is primarily based on expert opinion synthesized during advisory board discussions, it inherently possesses limitations related to the subjective nature of expert insights and the lack of large-scale randomized controlled trial data exclusively validating these precise recommendations. Further research is warranted to corroborate these findings in broader populations and diverse clinical settings.

In summary, this expert opinion offers a comprehensive, clinically relevant framework for managing AUB with NETA, balancing evidence with practical considerations, while acknowledging the need for ongoing research to address existing knowledge gaps.

## Conclusions

NETA delivers rapid and reliable control of abnormal uterine bleeding, with an established safety profile and strong patient adherence. With individualized dosing strategies, clinicians can tailor therapy effectively while minimizing side effects. Future large-scale studies will help refine optimal regimens and long-term outcomes. While based on expert opinion, these recommendations offer clear, actionable guidance and lay a strong foundation for future research to validate and refine optimal AUB care.
